# An Increasing Prominent Disease of *Klebsiella pneumoniae* Liver Abscess: Etiology, Diagnosis, and Treatment

**DOI:** 10.1155/2013/258514

**Published:** 2013-09-30

**Authors:** Yun Liu, Ji-yao Wang, Wei Jiang

**Affiliations:** Department of Gastroenterology, Zhongshan Hospital, Fudan University, Xuhui, Shanghai 200032, China

## Abstract

*Background*. During the past two decades, *Klebsiella pneumoniae* (*K. pneumoniae*) had surpassed *Escherichia coli* (*E. coli*) as the predominant isolate from patients with pyogenic liver abscess (PLA) in Asian countries, the United States, and Europe, and it tended to spread globally. Since the clinical symptom is atypical, the accurate and effective diagnosis and treatment of *K. pneumoniae* liver abscesses (KLAs) are very necessary. *Methods*. Here, we have comprehensively clarified the epidemiology and pathogenesis of KLA, put emphases on the clinical presentations especially the characteristic radiographic findings of KLA, and thoroughly elucidated the most effective antibiotic strategy of KLA. *Results*. K1 serotype is strongly associated with KLA especially in diabetic patients. Computed tomography (CT) and ultrasound (US) were two main diagnostic methods of KLA in the past. Most of KLAs have solitary, septal lobular abscesses in the right lobe of liver, and they are mainly monomicrobial. Broad-spectrum antibiotics combined with the US-guided percutaneous drainage of liver abscesses can increase their survival rates, but surgical intervention still has its irreplaceable position. *Conclusion*. The imaging features contribute to the early diagnosis, and the percutaneous intervention combined with an aminoglycoside plus either an extended-spectrum betalactam or a second- or third-generation cephalosporin is a timely and effective treatment of KLA.

## 1. Introduction

Pyogenic liver abscess (PLA) is a life-threatening infectious disease. Before the 1980s, *E. coli* was the most common pathogen that caused PLA and was mostly polymicrobial. However, during the past two decades, highly virulent strains of *K. pneumoniae* had emerged as a predominant cause of PLA in Asian countries and areas [1–5], the United States [6–11], and Europe [12–14], especially Taiwan [2, 15–22], and it tended to spread globally [23–26]. Recent researches have shown, unlike other bacterial-induced PLAs (Non-KLAs) which are mostly associated with biliary tract disorders [19, 27–30], that *K. pneumoniae* liver abscesses (KLAs) are often cryptogenic [3, 6, 17, 27, 29–34]. Metastatic meningitis or endophthalmitis is often complicated with KLA 10%–45% [9, 16, 30, 35–38], and most of KLA patients had diabetes mellitus [1, 15, 16, 22, 27, 29, 30, 35, 38, 39]. KLA has the characteristic radiographic findings which are different from those of Non-KLA [1, 6, 22, 30, 34, 38, 40, 41]. Up to date, the combination of systemic antibiotics and percutaneous drainage has become the treatment of choice for the management of KLA [9, 15, 16, 38, 42, 43].

In this paper, we comprehensively described the epidemiology and the pathogenesis of KLA. And then, we put emphases on the difference of clinical presentations especially the radiographic findings between KLA and other kinds of PLA (Non-KLA), in order to diagnose KLA early and precisely, and further elucidate the effective therapeutic methods of KLA especially the choice of targeted antibiotics. We hope to enhance the understanding of KLA and contribute to the timely, accurate, and effective diagnosis and treatment of this disease.

## 2. Epidemiology

In the recent two decades, *K. pneumoniae* has become the most common causative pathogen of PLA in Asian countries and areas [1–5, 15–22]. A shift from *E. coli* to *K. pneumoniae* as the causative pathogen of pyogenic liver abscess may also have occurred in the United States [6–11] and Europe [12–14]. However, differing from the much higher incidence of KLA in Asian countries and areas [1–5] especially the highest incidence in Taiwan that ranges from 80% to 90% [16, 19], a relatively low incidence of 30%–40% is estimated in the US [6, 9]. The exact cause for the increasing prevalence of KLA in Asia is not known, but it may be related to the large population of Asia, the host susceptibility to infection, the difference in carriage rates, and the environmental factors or the emergence of a distinct strain of *K. pneumonia,* and an increased propensity to cause liver abscesses might be potentially contributing to such a geographical difference in the epidemiology of *K. pneumonia* infection. For example, Chung et al. had noted that people of Korean ethnicity who had lived in countries other than Korea had a lower proportion of carrying serotype K1 of *K. pneumonia* strains than those who lived in Korea [3]. These findings indicate a potential role of the environmental factors in the intestinal colonization of these strains. 

The middle-to-older-aged patients are at higher risk of developing KLA. The peak incidence of the disease is 55–60 years old [4, 6, 7, 9, 16, 19, 40, 41]. Reports of KLA in children are rare [44]. Male dominance is found in patients with KLA, and the male-to-female ratio is approximately 1.5–2.5 : 1 [4, 7, 9, 14, 16, 19, 21, 35, 36, 38, 41] (Table 1).

The mortality rate of KLA is lower than that of the Non-KLA [16, 19, 35], ranging from 2% to 18% [6, 7, 15, 16, 18, 19, 35, 36, 38, 40, 46–48]. Owing to the improvement of diagnosis and people's awareness of treatment, the mortality rate decreases. 

## 3. Etiology

KLAs are usually primary and cryptogenic [3, 6, 17, 27, 29–34], and cryptogenic invasive KLAs are frequently associated with diabetes mellitus [22], but they does not show any clear association with peritoneal sources of infections, such as hepatobiliary obstruction, pancreatitis, enterocolitis, or malignant diseases. 

Some studies manifested that translocation from the gastrointestinal tract maybe the most likely route by which *K. pneumonia* caused liver abscess [3, 20]. Fung et al. demonstrated that gastrointestinal carriage was a predisposing factor for liver abscess [20]. Furthermore, they also found that patients with KLA and healthy carriers had identical pulsed-field gel electrophoresis (PFGE) profiles with the same virulence-associated genes and similar LD50 values. In a recent investigation, Lin et al. reported a fecal carriage rate of *K. pneumonia* in healthy adults of 75% and high prevalence (23%) of serotype K1/K2 isolates among typical strains in Taiwan [49]. *K. pneumonia* can colonize the gastrointestinal tract of humans, which suggests that colonization by the *K. pneumoniae* strains precedes invasion of the intestinal mucosa and portal venous flow or ascending biliary infection, which is followed by the development of liver abscess.

Several studies have found that capsule K1 and K2 were the most two common causes of KLA (Table 2). K1 isolates occur at a significantly higher frequency than those of all other serotypes, especially in patients with diabetes mellitus [15]. A recent case-control study performed by Kim et al. suggested that diabetes mellitus was an important underlying factor that correlates with a high incidence of K1 serotype in KLA [29]. Poor glycemic control plays an important role in impairing the neutrophil phagocytic function of patients with K1/K2-type KLA, whereas it does not significantly affect those of patients with non-K1/K2 KLA [50]. Capsule k1 serotype is found to express the hypermucoviscous phenotypes which can produce vast amounts of extracapsular polysaccharide constituting a mucoviscous web that protects these strains from phagocytosis by neutrophils and from serum killing by complement [2, 13, 51]. In addition, *in vitro* serum assays show a significantly higher serum resistance on average for K1 than K2 strains, indicating that K1 and K2 strains have unequal virulence [18]. Recently, most isolates of serotype K1 from KLA patients belong to ST23 which is the most prevalent sequence type among serotype K1 isolates [2, 24, 26].

## 4. Bacterial Genes and Pathogenesis

A number of bacterial genes, which are significantly correlated with the high virulence of the invasive strains [2, 24, 26, 52], have been proposed or suggested to play key roles in the pathogenesis of hepatovirulent KLA (Table 2). 

The* rmpA* gene (a regulator of the mucoid phenotype), which is a transcriptional activator of the cps genes and functions as a positive regulator of extracapsular polysaccharide synthesis, has a strong association with hypermucoid strains in PLA [18, 52, 53]. Loss of this regulator will downregulate capsule synthesis, and knockout of the *rmpA* gene can decrease virulence in mouse lethality tests by 1000 folds [12], leading to the loss of phagocytic resistance and the mucoid phenotype. Hsu et al. found a correlation of *rmpA*/*A2* with six PLA-related capsular types (K1, K2, K5, K54, K57, and KN1). However, the correlation of *rmpA/A2* with K1 strains from the West was less obvious than with the strains from Asia [53].

On the other hand, magA, a chromosomal gene which is located in the cps (capsular polysaccharide synthesis) operon, has been recently renamed wzyKpK1 and has been shown to be specific for K1 capsule formation [26]. MagA can contribute to capsular polysaccharide formation, and it is identified as an important virulence gene in invasive *K. pneumoniae* strains causing primary liver abscess and septic metastatic complications [54]. MagA mutants are also shown to lose the potential to produce this protective mucus and became susceptible to human serum and phagocytosis.

The growth of bacteria in host tissues is limited not only by host defense mechanisms but also by their supply of available iron. Many bacteria attempt to secure their supply of iron in the host by secreting high-affinity iron chelators called siderophores, like aerobactin. Aerobactin, an iron chelator called iron siderophore, can increase virulence in mouse lethality tests by 100 folds [55]. 

A 20 kb chromosomal region including an iron uptake system (kfu) and a phosphoenolpyruvate sugar phosphotransferase system (PTS) was found to be presented in most of the genomes of the tissue-invasive *K. pneumoniae* strains [18, 56]. Iron uptake is critical to pathogenesis as a vital cofactor for many components of microbial antioxidative stress defense. The kfu/PTS region could enrich the ability of bacteria to secure iron, even in the relatively iron-deficient conditions of the human host, and to eventually enhance the virulence of the bacteria. The celB gene, which encodes the putative cellobiose-specific PTS, has been confirmed to play an important role in the virulence of PLA-associated *K. pneumonia* strains. When deleting the celB, the PTS activity is significantly decreased, the biofilm development is delayed, and the thickness of the biofilm does not further increase [57].

The allS gene that possessed a 22 kb region associated with anaerobic metabolism of allantoin as the sole source of carbon, nitrogen, and energy under either aerobic or anaerobic condition [58] is only found in K1 isolates [52] (Table 2). The allS region can help bacteria to compete for nitrogen sources via the allantoin-utilizing ability. 

## 5. Clinical Manifestations

The presentations of KLA are not typical, and patients may present with vague constitutional symptoms. The relatively common presentation features are fever [7, 9, 13, 16, 26, 28, 35, 38, 41] and chills [7, 28, 35, 38, 41], followed by right abdominal pain [7, 16, 38]. Fever is predominant as the most common symptom and has been reported in 90%–95% of the cases [9, 16, 38] (Table 1). But there is also a broad array of nonspecific symptoms like diarrhea, jaundice, right pleural effusion [38], anorexia, nausea, and vomiting [9, 16]. Although the case of spontaneous rupture of a liver abscess has been rarely reported, there is a higher incidence of abscess rupture in the KLA patients than in Non-KLA patients [16, 27, 34, 38]. The risk factors for spontaneous rupture in KLA are diabetes mellitus, large abscess size, thined-wall abscess, and gas-forming abscess [34, 38, 59]. 

 KLA is also associated with a higher likelihood of hematogenous spread and the potential for metastatic infection in other parts of the body compared with other kinds of PLA [16, 21, 27, 30, 35, 38, 40, 41, 50, 56]. The high incidence rate of metastatic infection ranges from 10% to 45% [9, 16, 30, 35–38], especially in patients with diabetes mellitus [14, 15, 22, 30, 35, 38, 39, 50]. Patients with diabetes mellitus are at increased risk for common infection due to impaired host-defense mechanisms. Furthermore, abscess with size of 5-6 cm is proven to be a significant independent predictor of KLA patients with metastatic infections [22, 39]. It is suggested that we should not neglect the small-sized liver abscess in diabetic patients in the early course of the disease, for hematogenous dissemination of *K. pneumonia* can occur, which leads the severe clinical symptoms that can result in earlier detection of liver abscess, and this supported the metastatic infections of small-sized KLA.

 Eyes [9, 11, 13, 15, 30, 35, 36, 38], meninges [16, 35], CNS [16, 30], and lungs [9, 13, 16, 30] are the most common metastatic sites. Endophthalmitis is the most common and serious septic complication of KLA, leading to subacute vision impairment. These patients usually do not recover their vision and become legally blind despite aggressive intravenous and intravitreous antibiotics [13, 15, 35]. Recently, two studies indicated that the mortality rate of KLA patients with metastatic infections was significantly higher than that without metastatic infections (16%-17% versus 0%–1.1%) [22, 39].

 In terms of underlying diseases, individuals with KLA have a lower proportion of comorbidity such as malignancy [38], liver cirrhosis, chronic kidney disease, and biliary disease [30] than did Non-KLA ones, a significantly higher proportion of DM on the other hand [29, 33]. Other presentations such as bacteremia, septic shock, disseminated intravascular coagulation, acute renal failure, and acute respiratory failure are also reported to be more prevalent in KLA than in Non-KLA patients [38]. 

 On physical presentations, fever and right upper quadrant tenderness are the most common findings. Jaundice is found in the patients with underlying biliary disease. Hepatomegaly is less common in KLA than in Non-KLA patients [38]. 

## 6. Laboratory and Imaging Findings

Anemia, leukocytosis, high erythrocyte sedimentation rate, C-reactive protein, hypoalbuminemia, elevated total bilirubin, and alanine aminotransferase are the common features. A recent study in the USA found an elevated white blood cell count in 68%, a low albumin level in 70.2%, and an elevated alkaline phosphatase level in 67% of PLA [6]. None of the blood tests specifically helps to diagnose a liver abscess, but they can suggest a liver abnormality that leads to imaging studies.

The most essential technology to make the diagnosis of KLA is radiographic imaging. Pulmonary X-ray can reveal right-sided pulmonary infiltrates with pleural effusion [34, 60], and plain abdominal X-rays, which are rarely used but can be helpful in some cases, can show air-fluid levels [45, 59] or portal venous gas. And now, US and CT are two main diagnostic methods which are both sensitive in the diagnosis of KLA.

The appearance of KLA at US imaging may range from hyperechoic to hypoechoic, and this variation has a close relationship to the pathologic stage of KLA. Hui et al. found that 84% patients predominantly had a solid appearance unlike other PLAs in US imaging and that 52% patients had diabetes mellitus [40]. However, Lee et al. found that KLA predominantly had a solid appearance but did not show much association with diabetes mellitus (the prevalence of diabetes mellitus between KLA and Non-KLA is 61.0% versus 51.4%, resp.) [30]. Overall, KLA appears as hypoechoic nodules and solid in US appearance imaging.

**Figure 1:**
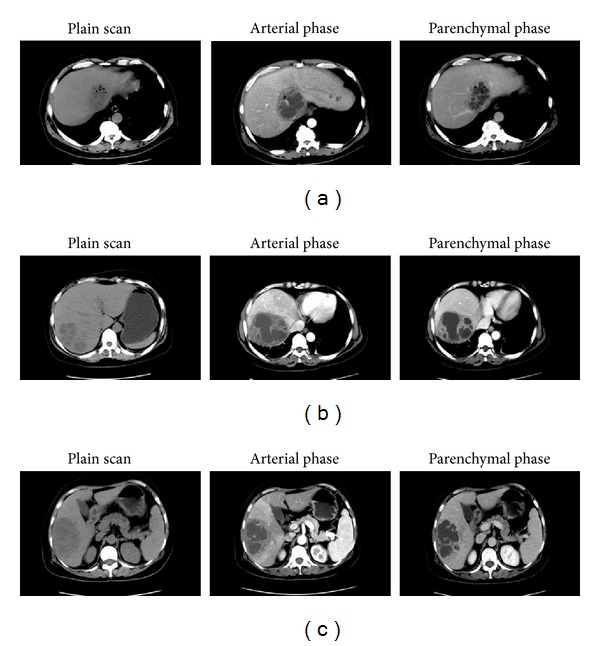
Comparison of abdominal CT images between KLA and Non-KLA.  (a) CT images of a 57-year-old male KLA patient with concomitant diabetes mellitus: circular shadow of low and uneven density can be seen in the caudate lobe near the second hepatic portal. With a diameter of 90 mm, a shadow of much lower density and gas cavities can be seen in the center of the abscess. During enhanced scanning, the margin and internal septations of abscess show a honeycomb-like structure. Intrahepatic bile ducts show slight dilation. (b) CT images of a 51-year-old female patient with *E. coli* liver abscess: irregular low-density lesion with a honeycomb-like structure can be seen in the right lobe of the liver. Obvious cystic wall enhancement can be seen during enhanced scanning. There is no stenosis or filling defect of hepatic vessels. (c) CT images of a 65-year-old female patient with *Pseudomonas aeruginosa* liver abscess: patchy shadow of low-and-even density and clear edge can be seen in the right lobe of the liver. By enhanced CT scan, the peripheral enhancement is more dramatic than the nonperipheral enhancement. Septation is visible inside the abscess, and hepatic blood vessels are evenly distributed. KLA: *Klebsiella pneumoniae* liver abscess; Non-KLA: *non-Klebsiella pneumonia*-induced pyogenic liver abscess.

 The sensitivity of CT was reported to reach as high as 100% compared with a sensitivity of 96% of US [47]. Recently, various studies were reported to compare the differences of CT imaging between KLA and Non-KLA [1, 27, 30, 38, 41] (Table 3). Most of KLA patients have a solitary abscess in the right lobe of liver due to its size and propensity to receive most of the portal blood flow [1, 6, 9, 27, 28, 30, 35, 38, 40, 41], which does not show much difference from Non-KLA ones [1, 27, 30, 38]. However, some reports found that KLAs were more likely to appear as single abscesses [35, 41] and unilobar involvements [41] than Non-KLAs (unilobar: 82.6% versus 61.5%). The majority of liver abscesses in the two groups are not more than 10 cm in diameter, and there are no significant differences between KLA and Non-KLA with respect to the size of the abscess cavity [1, 35, 38, 41]. KLA is predominantly with septations in the abscess (i.e., multilocular), which is similar to that of Non-KLA [30, 38, 41], whereas multilocular abscesses are more common in the KLA group than in the Non-KLA group [41]. Gas-forming liver abscess had been rarely reported in pyogenic liver abscess in the past; however, with the etiologic shift to *K. pneumoniae* as the primary causative agent of PLA infections, there is an increased risk of gas-producing liver abscess especially in patients with uncontrolled DM [1, 5, 22, 45, 60]. In our previous research, we found that KLA was more associated with gas-formation than Non-KLA [24(32.9%) versus 5 (13.5%)] [1] (Figure 1). It is assumed that, under anaerobic conditions, these facultative anaerobes can produce carbon dioxide by fermentation of glucose in tissue, especially under hyperglycemic conditions. No differences are found between groups regarding the presence of gas bubbles [30, 35, 38, 41] as shown in Table 3; however, KLA also shows a trend toward higher incidence of thrombophlebitis, whereas pneumobilia is more common in the Non-KLA group [41]. Other series do not show much difference between KLA and Non-KLA on account of the incidence of pneumobilia and thrombophlebitis [1, 38].

On CT imaging, liver abscesses are of lower attenuation than the surrounding normal liver parenchyma, thin-walled abscess on unenhanced scans [30, 34, 38]. The abscess wall usually shows a rim-enhancement on contrast-enhanced CT [38].

## 7. Therapy

### 7.1. Antibiotics Medication

When the diagnosis of KLA is suspected, broad-spectrum antibiotics are started immediately to control ongoing bacteremia and its associated complications. Many studies have found that most isolates are resistant to ampicillin [7, 9, 15–17, 26, 29, 51] with an MIC90 of 32 mg/mL [17] and penicillin [9, 29], but susceptible to third- and fourth-generation cephalosporins, quinolones, aminoglycosides, and carbapenems [4, 15–17, 38, 61]. Cephalosporins have a dominant position in antibiotic treatment of KLA [4, 38, 62] (Table 4).

 Although a significantly higher complication rate is found in KLA patients treated with cefazolin than in those treated with an extended-spectrum cephalosporin [63], another research has shown the similar therapeutic effects between patients treated with extended-spectrum cephalosporins and those treated with a combination of first-generation cephalosporins and aminoglycosides; furthermore, the latter treatment is recommended for patients without risk factors such as endophthalmitis and meningitis [62] (Table 4). 

The third-generation cephalosporin is used more in patients with KLA group as compared with that in the *Streptococcus milleri* (SM) group [38]. The SM group tends to use extended-spectrum penicillin more, whereas 10% of *Klebsiella* isolates are resistant to penicillin. Initial antibiotic regimens should comprise a second-generation cephalosporin and an aminoglycoside with metronidazole when treating PLA caused by *E. coli* isolates, whereas the first-generation cephalosporin covers most pathogens found in KLA [27]. 

The optimal duration intravenous therapy, as well as the duration of subsequent oral therapy, remains unclear. In the study of Taiwan, therapy generally consisted of 3 weeks of intravenous antibiotics followed by 1-2 months of oral therapy [16]. However, a US study in 2004 indicated shorter courses of antibiotic therapy with durations of intravenous therapy (17.5 days), and oral therapy (13.6 days) which were associated with extremely low mortality [6]. 

The treatment of KLA without metastatic infections includes pigtail catheter drainage by negative-pressure and combination of parenteral cefazolin and gentamicin for two weeks [16]. Gentamicin is discontinued after 2 weeks to avoid nephrotoxicity, but cefazolin is continued for at least 3 weeks and oral cephalosporin for 1-2 months to prevent relapse. In patients with septic endophthalmitis or other distal metastases, the prognosis is bad. Systemic intravenous and intravitreous antibiotics are necessary [35, 64, 65]. For example, a third-generation cephalosporin, ceftriaxone, is considered to be a useful antibiotic due to its good penetration into the vitreous compartment [15]. Intravitreal vancomycin and ceftazidime are also successfully administrated in some studies [64, 65].

 Resistance of *K. pneumonia* to strains that produce extended-spectrum *β*-lactamase (ESBL) had been noted in many parts of the world [6, 9, 66]. Antibiotics such as *β*-lactam/ for Beta-lactamase inhibitor combinations such as piperacillin/tazobactam (TZP) and ampicillin/sulbactam have replaced extended-spectrum cephalosporins (ESCs) to control ESBL prevalence in Korea [67]. Recently, TZP resistance among *K. pneumonia* isolates has been shown as high as 20.9% (50/239) versus 7.6% (13/170) of *E. coli*, and the mechanisms for TZP resistance might include the presence of AmpC producers, multiple b-lactamases in individual organisms of a given isolate, and possible TEM-1 hyperproducers [66]. Drug-resistant *K. pneumonia *is more prevalent in the DM group than in the non-DM group [68]. If a patient presents with the risk factors for infection with ESBL-producing organisms, carbapenem antibiotics (e.g., imipenem, meropenem, ertapenem, or doripenem) should be recommended before the culture and isolation results are obtained. Carbapenem (primarily imipenem) has been found to be independently associated with lower mortality than other antibiotics [61]. 

### 7.2. Interventional Therapy

Percutaneous drainage was widely used during the past two decades [6, 37, 46, 69], and the obvious advantages are the simplicity of treatment and avoidance of general anesthesia and laparotomy. As for multiple abscesses, percutaneous drainage usually meets with a higher failure rate [47]. However, a recent retrospective study showed that the treatment with percutaneous transhepatic drainage demonstrated similar effectiveness for the patients with multiple abscesses but shorter hospitalization when compared with surgical drainage group, which suggested that percutaneous drainage should always be undertaken before surgery in terms of its lower morbidity and less cost [69]. 

Percutaneous drainage includes percutaneous aspiration and percutaneous catheter drainage. Yu et al. found that intermittent needle aspiration was probably as effective as continuous catheter drainage for the treatment of PLA [46]. Due to the solid nature of KLA, procedure simplicity, patient comfort, and reduced price, needle aspiration deserves to be considered as a first-line drainage approach. Patients with the following criteria are taken for percutaneous drainage: (1) patients who continued to be febrile even after 48–72 h of adequate medical treatment; (2) liver abscess more than 6 cm in size; and (3) clinical or ultrasonographic features suggesting impending perforation [70]. 

### 7.3. Surgical Intervention

Although percutaneous drainage has replaced surgery as the primary treatment of liver abscess [37, 69], the surgery still has its irreplaceable position under some conditions. Surgery drainage is carried out in patients falling within the criteria which are as follows: (1) thick pus which could not be aspirated; (2) patients with multiple liver abscess; (3) patients with ongoing sepsis even after antibiotic therapy and percutaneous drainage; (4) patients with underlying diseases such as the biliary tract disease or the liver cirrhosis; (5) multiloculated abscess; (6) abscess in the left lobe; and (7) ruptured abscesses [28, 34, 36, 37, 59, 60, 69, 71, 72]. 

Recently, a case with intraperitoneal rupture of pyogenic liver abscess caused by *K. pneumonia* was successfully treated with hepatectomy combined with antibiotics [34]. The solid nature of the abscess and the complicating DIC preclude percutaneous catheter drainage of the abscess. This indicates that surgical hepatectomy is necessary and useful in KLA treatment. In general, surgical drainage has been reserved for patients who fail to respond to treatment with percutaneous drainage and antibiotics or who have concurrent intra-abdominal pathology which requires surgical management [47]. Thus, percutaneous and surgical techniques are not competing methods, but they have different indications, and surgery also represents an option for nonresponders to percutaneous treatment.

### 7.4. Glycemic Control

Furthermore, glycemic control in diabetic patients plays an essential role in the clinical features of KLA, especially in metastatic complications from KLA [49, 50]. 

## 8. Prognosis

Overall, the prognosis is better for patients with KLA than for those with other bacterial liver abscesses insofar as mortality [16, 19, 35] and disease relapse [16]. Diabetes mellitus and the K1 serotype were common risk factors for recurrent KLA [51, 73]. The outlook for patients who develop metastatic infections especially endophthalmitis is grim [13, 15, 16]. 

## 9. Conclusion

PLA caused by *K. pneumonia *is an emerging infectious disease in diabetic patients in Asian countries and areas, the United States, and Europe, and it tends to spread globally. Strains of capsule K1 are the most virulent serotypes and are commonly associated with KLA and its complications. Various genes contribute to these hypermucoviscous features, including rmpA, aerobactin, magA, kfu, and allS, which can be exploited as a genetic marker for rapid molecular diagnosis and for treatment of this disease. The characteristics of imaging features contribute to the early diagnosis, and percutaneous intervention combined with an aminoglycoside plus either an extended-spectrum betalactam, such as piperacillin/sulbactam or a second- or third-generation cephalosporin, is a timely and effective treatment of KLA. The prognosis is better for patients with KLA than for those with other bacterial liver abscesses; however, KLAs with metastatic infections especially endophthalmitis have poor outcomes.

## Figures and Tables

**Table 1:** Demographic, clinical characteristics and treatment of patients with *Klebsiella pneumoniae* liver abscesses from case reports.

Case	Age	Sex	Race	Underlying diseases	Symptom	Location of medical therapy		Outcome	Reference
1	64	F	Filipino	Diabetes mellitus thrombocytosis	Fever, rigors, nausea, and myalgias	Right lobe	Piperacilin-tazobactam (3.37 g Q6h) + gentamicin (400 mg qd), ceftriaxone (2 g iv qd) + oralmetronidazole (500 mg iv four times daily) followed by levofloxacin and metronidazole for 4 wks	Survived	[7]
2	71	M	Caucasian	Coronary artery disease	Fever, abdominal pain, and hypotension	Left lobe	Cefotetan (2 g BID) + oral levofloxacin (500 mg qd) for 8 wks	Survived	[7]
3	53	M	Caucasian	Mitral valve prolapse and hypercholesterolemia	Fever, rigors, fatigue, malaise, night sweats, and tooth pain	Left lobe	Ceftriaxone + metronidazole for 4 wks, gentamicin for 2 wks, followed by oral ciprofloxacin for 1 month	Survived	[7]
4	64	F	Filipino	Peptic ulcer disease, coronary artery disease, and hypertension	Fever, right abdominal pain, and anorexia	Left lobe	Ciprofloxacin (400 mg, iv, bid) + metronidazole (500 mg iv, tid), followed by oral ciprofloxacin + metronidazole for 6 wks	Survived	[7]
5	56	M	Filipino	None	Fever, chills, night sweats, epigastric pain, and nausea	Right lobe	Piperacillin/tazobactam (q6h) + metronidazole (500 mg, q8h), gentamicin (180 mg, q18h), followed by oral levofloxacin (500 mg, qd) + metronidazole (500 mg, tid) for 6 wks	Survived	[7]
6	59	F	Filipino	Diabetes mellitus	Fever, chills, anorexia, and fatigue	Left lobe	Piperacillin/tazobactam (3.375 g, q6h) + metronidazole (500 mg, q8h), ceftriaxone (2 g/day) + metronidazole (500 mg, q8h) for 4 wks, followed by oral levofloxacin (500 mg/day) for 3 months	Survived	[7]
7	55	M	Argentinian	None	Fever and fatigue	NR	Ceftriaxone + metronidazole, followed by oral ciprofloxacin for 6 wks, percutaneous drainage	Survived	[26]
8	47	F	Omani	None	Fever, chills, rigors, mild cough, poor oral intake, and inability to walk	Right lobe	Augmentin (2 g iv q6h)+ gentamicin (1.7 g IV q8h) for 3 wks, catheter drainage	Survived	[18]
9	58	F	Omani	Diabetes millitus	Fever, malaise, and nausea	Right lobe	Amikacin (1 g IV q12h) + ceftazidime (1.5 g IV q8h for 5 days), followed by ciprofloxacin (0.5 g IV q12h for 12 days) and piperacillin/tazobactam (4.5 g iv q8h) for 15 days	Survived	[18]
10	62	M	Irish	Peripheral vascular disease and excess alcohol intake	Abdominal pain, anorexia and nausea	NR	Piperacillin/tazobactam (4.5 g iv q8h) for 15 days	Survived	[14]
11	40	M	Filipino	Diabetes mellitus	Fever, polydipsia, and polyuria	Right lobe	Ceftriaxone (2 g iv qd), oralciprofloxacin for 69 days; percutaneous drainage, intravenous gentamicin, and ciprofloxacin (400 mg, iv bid) for 5 days	Survived	[14]
12	55	M	Chinese	Diabetes mellitus	Vomiting, abdominal pain, fever, and rigors	Right lobe	Oral ciprofloxacin for 36 days, oral cephalexin for 97 days, amoxicillin-clavulanic acid (1.2 g, iv, tid) + gentamicin (320 mg/day) + metronidazole (500 mg, iv, tid), followed by ceftriaxone (2 g/day·iv) + oral metronidazole (400 mg, bid), percutaneous drainage	Survived	[14]
13	58	M	Japanese	Diabetes mellitus	Malaise	Right lobe	Meropenem (1 g iv q12h) for 6 days, cefmetazole (2 g iv Q8h), oral cefcapene pivoxil (100 mg, tid), and antibiotic treatment for 30 days	Survived	[5]
14	61	F	Japanese	Diabetes mellitus	Fever, chills, and a slight headache	NR	Meropenem (1 g, iv, q8h) + linezolid (600 mg, iv, q12h), changed to ceftriaxone (2 g q12h) for 20 days, followed by oral cephalexin (250 mg q6h) for 31 days	Survived	[5]
15	43	M	Japanese	None	Right hypochondriac and epigastric pain	Right lobe	Meropenem (1 g day iv) for 15 days + IV insulin, mg/day, and oral ciprofloxacin (400 mg/day) for 50 days	Survived	[45]

NR: not reported.

**Table 2:** Genes associated with the serotypes of *K*. *pneumonia*.

Gene	Comment	K1	K2	Non-K1/K2	Reference
MagA	Capsular polysaccharide synthesis	+	§	§	[24, 26]
RmpA	Regulator of the mucoid phenotype	+	+	+	[2, 26, 52]
kfu/PTS	Iron uptake system (kfu) and a phosphoenolpyruvate	+	−	+	[52]
Aerobactin	An iron chelator	+	+	+	[2, 26, 52]
AllS	Anaerobic metabolism of allantoin	+	−	−	[52]
No. of isolates (%) (*n* = 248)	—	63.40%	14.20%	22.40%	[15]

§: No data; −: lack of this gene.

**Table 3:** Comparison of CT imaging characteristics between KLA and non-KLA reported from Hong Kong (38), Korea (30), Singapore (41), Taiwan (35), and China (1).

Parameters	Hong Kong (38) (*n* = 161)			Korea (30) (*n* = 129)			Singapore (41) (*n* = 131)			China (1) (*n* = 110)			Taiwan (35) (*n* = 248)		
	KLA (140)	Non-KLA (21)	*P* value	KLA (59)	Non-KLA (70)	*P* value	KLA (92)	Non-KLA (39)	*P* value	KLA (73)	Non-KLA (37)	*P* value	KLA (171)	Non-KLA (77)	*P* value
No. of abscess									0.01						<0.05
Solitary (*n* = 1)	—	—	—	—	—	—	73 (79.3%)	22 (56.4%)		60 (82.2%)	30 (81.1%)	NS	125 (73.1%)	45 (58.4%)	
Multiple (*n* > 1)	—	—	—	—	—	—	19 (20.7%)	17 (43.6%)		13 (17.8%)	7 (18.9%)	NS	46 (26.9%)	32 (41.6%)	
Location						0.312			0.01						NS
Right	97 (69.3%)	15 (71.4%)	0.42	41 (69.5%)	49 (70.0%)		76 (82.6%)^§^	(61.5%)^§^		47 (64.4%)	24 (64.9%)*	NS	128 (74.9%)	52 (67.5%)	
Left	31 (22.1%)	4 (19.1%)	0.38	14 (23.7%)	16 (22.9%)		^§^	^§^		11 (15.1%)	5 (16.7%)*	NS	34 (19.9%)	19 (24.7%)	
Both	12 (8.6%)	2 (9.5%)	0.44	4 (6.8%)	5 (7.1%)		16 (17.4%)	15 (38.5%)		2 (2.7%)*	1 (2.7%)*	NS	9 (5.2%)	6 (7.8%)	
													<5,72 (42.1%);	<5,36 (46.8%);	NS
Size (cm)	6.5 ± 2.8^#^	7.4 ± 2.9^#^	0.19	—	—	—	7.3 ± 2.8^#^	7.8 ± 2.8^#^	0.35	7.4 ± 2.4^#^	7.4 ± 3.2^#^	NS	5–10,87 (50.9%)	5–10,37 (48.1%)	
Septations within abscess						0.103			0.01						—
Unilocular	—	—	—	9 (15.3%)	19 (27.1%)		5 (5.4%)	11 (28.2%)		—	—	—	—	—	
Multilocular	84 (60%)	13 (61.9%)	0.43	50 (84.7%)	51 (72.9%)		87 (94.6%)	28 (71.8%)		41 (38.7%)	20 (35.7%)	NS	—	—	
Gas-formation in abscess	13 (9.3%)	2 (9.5%)	0.49	52 (89.7%)	64 (91.4%)	0.536	11 (28.2%)	6 (15.4%)	0.58	24 (32.9%)	5 (13.5%)	<0.05	7 (4.1%)	4 (5.2%)	NS
Septal enhancement	—	—	—	44 (74.6%)	41 (58.6%)	0.056	—	—	—	30 (41.1%)	6 (16.2%)	<0.05	—	—	—
Rim-enhancement	68 (48.6%)	12 (57.1%)	0.23	20 (33.9%)	43 (61.4%)	0.004	—	—	—	28 (38.4%)	12 (32.4%)	NS	—	—	—
Pneumobilia	9 (6.4%)	0 (0.0%)	0.12	—	—	—	1 (1.1%)	5 (12.8%)	0.01	7 (9.6%)	3 (8.1%)	NS	—	—	—
Thrombophlebitis	2 (1.4%)	1 (4.8%)	0.13	—	—	—	28 (30.4%)	2 (5.1%)	<0.01	—	—	—	—	—	—

—: There were no data in these references; ^§^locations of right and left are not mentioned separately in reference [41]; ^#^means ± standard; *there were other locations of abscess in addition to those mentioned in reference [1].

**Table 4:** Antibiotic treatment in patients with *Klebsiella  pneumoniae* liver abscess and *Streptococcus  milleri* liver abscess.

	KLA								*Streptococcus milleri *	
	Hong Kong (38) (*n* = 140)		Singapore (4) (*n* = 109)		Taiwan (62) (*n* = 110)		Turkey (61) (*n* = 85)		Hong Kong (38) (*n* = 21)	
	Duration (days)	Efficiency	Duration (days)	Efficiency	Duration (days)	Efficiency	Duration (days)	Efficiency	Duration (days)	Efficiency
Extended-spectrum penicillin*	21.7	48 (34.3%)	—	—	—	—	—	4 (4.7%)	15.4	11 (52.4%)
First- and second-generation cephalosporins^§^	21.7	30 (21.4%)	32 ± 13	24 (22.0%)	—	104 (94.5%)	—	—	15.4	4 (19%)
Third- and fourth-generation cephalosporins^▲^	21.7	50 (35.7%)	32 ± 13	71 (65.1%)	—	4 (3.6%)	—	5 (5.9%)	15.4	3 (14.3%)
Carbapenems^*∧*^	—	—	32 ± 13	13 (11.9%)	—	—	—	42 (49.4%)	—	—
Aminoglycosides^#^	—	—	—	1 (0.9%)	—	104 (94.5%)	—	2 (2.4%)	—	—
Quinolone**	—	—	—	—	—	—	—	11 (12.9%)	—	—

*Piperacillin-tazobactam, ticarcillin-clavulanate; ^§^cefazolin; ^▲^ceftriaxone, cefepime; ^∧^ertapenem, meropenem; ^#^amikacin; **ciprofloxacin.

## References

[1] Li J, Fu Y, Wang JY (2010). Early diagnosis and therapeutic choice of *Klebsiella pneumoniae* liver abscess. *Frontiers of Medicine in China*.

[2] Siu LK, Fung C, Chang F (2011). Molecular typing and virulence analysis of serotype K1 *Klebsiella pneumoniae* strains isolated from liver abscess patients and stool samples from noninfectious subjects in Hong Kong, Singapore, and Taiwan. *Journal of Clinical Microbiology*.

[3] Chung DR, Lee H, Park MH (2011). Fecal carriage of serotype K1 *Klebsiella pneumoniae* ST23 strains closely related to liver abscess isolates in Koreans living in Korea. *European Journal of Clinical Microbiology and Infectious Diseases*.

[4] Chan DS, Archuleta S, Llorin RM, Lye DC, Fisher D (2013). Standardized outpatient management of *Klebsiella pneumoniae* liver abscesses. *International Journal of Infectious Diseases*.

[5] Hagiya H, Kuroe Y, Nojima H (2013). Emphysematous liver abscesses complicated by septic pulmonary emboli in patients with diabetes: two cases. *Internal Medicine*.

[6] Rahimian J, Wilson T, Oram V, Holzman RS (2004). Pyogenic liver abscess: recent trends in etiology and mortality. *Clinical Infectious Diseases*.

[7] Lederman ER, Crum NF (2005). Pyogenic liver abscess with a focus on *Klebsiella pneumoniae* as a primary pathogen: an emerging disease with unique clinical characteristics. *The American Journal of Gastroenterology*.

[8] Golia P, Sadler M (2006). Pyogenic liver abscess: Klebsiella as an emerging pathogen. *Emergency Radiology*.

[9] Pastagia M, Arumugam V (2008). *Klebsiella pneumoniae* liver abscesses in a public hospital in Queens, New York. *Travel Medicine and Infectious Disease*.

[10] Pope JV, Teich DL, Clardy P, McGillicuddy DC (2011). *Klebsiella pneumoniae* liver abscess: an emerging problem in North America. *Journal of Emergency Medicine*.

[11] Sachdev DD, Yin MT, Horowitz JD, Mukkamala SK, Lee SE, Ratner AJ (2013). *Klebsiella pneumoniae* K1 liver abscess and septic endophthalmitis in a U.S. resident. *Journal of Clinical Microbiology*.

[12] Nassif X, Fournier JM, Arondel J, Sansonetti PJ (1989). Mucoid phenotype of *Klebsiella pneumoniae* is a plasmid-encoded virulence factor. *Infection and Immunity*.

[13] Sobirk SK, Struve C, Jacobsson SG (2010). Primary *Klebsiella pneumoniae* liver abscess with metastatic spread to lung and eye, a North-european case report of an emerging syndrome. *Open Microbiology Journal*.

[14] Moore R, 'Shea DO, Geoghegan T, Mallon PW, Sheehan G (2013). Community-acquired *Klebsiella pneumoniae* liver abscess: an emerging infection in Ireland and Europe. *Infection*.

[15] Fung CP, Chang FY, Lee SC (2002). A global emerging disease of *Klebsiella pneumoniae* liver abscess: Is serotype K1 an important factor for complicated endophthalmitis?. *Gut*.

[16] Wang JH, Liu Y, Lee SS (1998). Primary liver abscess due to *Klebsiella pneumoniae* in Taiwan. *Clinical Infectious Diseases*.

[17] Chang SC, Fang CT, Hsueh PR, Chen YC, Luh KT (2000). *Klebsiella pneumoniae* isolates causing liver abscess in Taiwan. *Diagnostic Microbiology and Infectious Disease*.

[18] Fang CT, Lai SY, Yi WC, Hsueh PR, Liu KL, Chang SC (2007). *Klebsiella pneumoniae* genotype K1: an emerging pathogen that causes septic ocular or central nervous system complications from pyogenic liver abscess. *Clinical Infectious Diseases*.

[19] Tsai FC, Huang YT, Chang LY, Wang JT (2008). Pyogenic liver abscess as endemic disease, Taiwan. *Emerging Infectious Diseases*.

[20] Fung CP, Lin YT, Lin JC (2012). *Klebsiella pneumoniae* in gastrointestinal tract and pyogenic liver abscess. *Emerging Infectious Diseases*.

[21] Keller JJ, Tsai MC, Lin CC, Lin YC, Lin HC (2013). Risk of infections subsequent to pyogenic liver abscess: a nationwide population-based study. *Clinical Microbiology and Infection*.

[22] Lin YT, Wang FD, Wu PF, Fung CP (2013). *Klebsiella pneumoniae* liver abscess in diabetic patients: association of glycemic control with the clinical characteristics. *BMC Infectious Diseases*.

[23] Keynan Y, Karlowsky JA, Walus T, Rubinstein E (2007). Pyogenic liver abscess caused by hypermucoviscous *Klebsiella pneumoniae*. *Scandinavian Journal of Infectious Diseases*.

[24] Turton JF, Englender H, Gabriel SN, Turton SE, Kaufmann ME, Pitt TL (2007). Genetically similar isolates of *Klebsiella pneumoniae* serotype K1 causing liver abscesses in three continents. *Journal of Medical Microbiology*.

[25] Pang TCY, Fung T, Samra J, Hugh TJ, Smith RC (2011). Pyogenic liver abscess: an audit of 10 years’ experience. *World Journal of Gastroenterology*.

[26] Vila A, Cassata A, Pagella H (2011). Appearance of *Klebsiella pneumoniae* liver abscess syndrome in Argentina: case report and review of molecular mechanisms of pathogenesis. *Open Microbiology Journal*.

[27] Chen SC, Wu W, Yeh CH (2007). Comparison of *Escherichia coli* and *Klebsiella pneumoniae* liver abscesses. *The American Journal of the Medical Sciences*.

[28] Mischinger HJ, Hauser H, Rabl H (1994). Pyogenic liver abscess: studies of therapy and analysis of risk factors. *World Journal of Surgery*.

[29] Kim JK, Chung DR, Wie SH, Yoo JH, Park SW (2009). Risk factor analysis of invasive liver abscess caused by the K1 serotype *Klebsiella pneumoniae*. *European Journal of Clinical Microbiology and Infectious Diseases*.

[30] Lee NK, Kim S, Lee JW (2011). CT differentiation of pyogenic liver abscesses caused by *Klebsiella pneumoniae* versus non-*Klebsiella pneumoniae*. *The British Journal of Radiology*.

[31] Chen SC, Huang CC, Tsai SJ (2009). Severity of disease as main predictor for mortality in patients with pyogenic liver abscess. *The American Journal of Surgery*.

[32] Fierer J (2012). Biofilm formation and *Klebsiella pneumoniae* liver abscess: true, true and unrelated?. *Virulence*.

[33] Huang WK, Chang JW, See LC (2012). Higher rate of colorectal cancer among patients with pyogenic liver abscess with *Klebsiella pneumoniae* than those without: an 11-year follow-up study. *Colorectal Disease*.

[34] Morii K, Kashihara A, Miura S (2012). Successful hepatectomy for intraperitoneal rupture of pyogenic liver abscess caused by *Klebsiella pneumoniae*. *Clinical Journal of Gastroenterology*.

[35] Yang CC, Yen CH, Ho MW, Wang JH (2004). Comparison of pyogenic liver abscess caused by non-*Klebsiella pneumoniae* and *Klebsiella pneumoniae*. *Journal of Microbiology, Immunology and Infection*.

[36] Basu S (2009). *Klebsiella pneumoniae*: an emerging pathogen of pyogenic liver abscess. *Oman Medical Journal*.

[37] Mezhir JJ, Fong Y, Jacks LM (2010). Current management of pyogenic liver abscess: surgery is now second-line treatment. *Journal of the American College of Surgeons*.

[38] Law ST, Li MKK (2013). Is there any difference in pyogenic liver abscess caused by *Streptococcus milleri* and *Klebsiella* spp?: Retrospective analysis over a 10-year period in a regional hospital. *Journal of Microbiology, Immunology and Infection*.

[39] Shin SU, Park CM, Lee Y, Kim EC, Kim SJ, Goo JM (2013). Clinical and radiological features of invasive *Klebsiella pneumoniae* liver abscess syndrome. *Acta Radiologica*.

[40] Hui JY, Yang MKW, Cho DHY (2007). Pyogenic liver abscesses caused by *Klebsiella pneumoniae*: US appearance and aspiration findings. *Radiology*.

[41] Alsaif HS, Venkatesh SK, Chan DSG, Archuleta S (2011). CT appearance of pyogenic liver abscesses caused by *Klebsiella pneumoniae*. *Radiology*.

[42] Anstey JR, Fazio TN, Gordon DL (2010). Community-acquired *Klebsiella pneumoniae* liver abscesses—an “emerging disease” in Australia. *Medical Journal of Australia*.

[43] Su YJ, Lai YC, Lin YC, Yeh YH (2010). Treatment and prognosis of pyogenic liver abscess. *International Journal of Emergency Medicine*.

[44] El-Shabrawi M, Hassanin F (2012). Pyogenic liver abscess. *Textbook of Clinical Pediatrics*.

[45] Tatsuta T, Wada T, Chinda D (2011). A case of gas-forming liver abscess with diabetes mellitus. *Internal Medicine*.

[46] Yu SCH, Ho SSM, Lau WY (2004). Treatment of pyogenic liver abscess: prospective randomized comparison of catheter drainage and needle aspiration. *Hepatology*.

[47] Malik AA, Bari SU, Rouf KA, Wani KA (2010). Pyogenic liver abscess: changing patterns in approach. *World Journal of Gastrointestinal Surgery*.

[48] Siu LK, Yeh KM, Lin JC, Fung CP, Chang FY (2012). *Klebsiella pneumoniae* liver abscess: a new invasive syndrome. *The Lancet Infectious Diseases*.

[49] Lin YT, Siu LK, Lin JC (2012). Seroepidemiology of *Klebsiella pneumoniae* colonizing the intestinal tract of healthy Chinese and overseas Chinese adults in Asian countries. *BMC Microbiology*.

[50] Lin JC, Siu LK, Fung CP (2006). Impaired phagocytosis of capsular serotypes K1 or K2 *Klebsiella pneumoniae* in type 2 diabetes mellitus patients with poor glycemic control. *Journal of Clinical Endocrinology and Metabolism*.

[51] Yang YS, Siu LK, Yeh KM (2009). Recurrent *Klebsiella pneumoniae* liver abscess: clinical and microbiological characteristics. *Journal of Clinical Microbiology*.

[52] Yu WL, Ko WC, Cheng KC, Lee CC, Lai CC, Chuang YC (2008). Comparison of prevalence of virulence factors for *Klebsiella pneumoniae* liver abscesses between isolates with capsular K1/K2 and non-K1/K2 serotypes. *Diagnostic Microbiology and Infectious Disease*.

[53] Hsu CR, Lin TL, Chen YC, Chou HC, Wang JT (2011). The role of *Klebsiella pneumoniae* rmpA in capsular polysaccharide synthesis and virulence revisited. *Microbiology*.

[54] Fang CT, Chuang YP, Shun CT, Chang SC, Wang JT (2004). A novel virulence gene in *Klebsiella pneumoniae* strains causing primary liver abscess and septic metastatic complications. *Journal of Experimental Medicine*.

[55] Nassif X, Sansonetti PJ (1986). Correlation of the virulence of *Klebsiella pneumoniae* K1 and K2 with the presence of a plasmid encoding aerobactin. *Infection and Immunity*.

[56] Ma LC, Fang C, Lee CZ, Shun CT, Wang JT (2005). Genomic heterogeneity in *Klebsiella pneumoniae* strains is associated with primary pyogenic liver abscess and metastatic infection. *Journal of Infectious Diseases*.

[57] Wu MC, Chen YC, Lin TL, Hsieh PF, Wang JT (2012). Cellobiose-specific phosphotransferase system of *Klebsiella pneumoniae* and its importance in biofilm formation and virulence. *Infection and Immunity*.

[58] Chou HC, Lee CZ, Ma LC, Fang CT, Chang SC, Wang JT (2004). Isolation of a chromosomal region of *Klebsiella pneumoniae* associated with allantoin metabolism and liver infection. *Infection and Immunity*.

[59] Ukikusa M, Inomoto T, Kitai T (2001). Pneumoperitoneum following the spontaneous rupture of a gas-containing pyogenic liver abscess: report of a case. *Surgery Today*.

[60] Alvarez Pérez JA, González JJ, Baldonedo RF (2001). Clinical course, treatment, and multivariate analysis of risk factors for pyogenic liver abscess. *The American Journal of Surgery*.

[61] Paterson DL, Ko W, von Gottberg A (2004). Antibiotic therapy for *Klebsiella pneumoniae* bacteremia: implications of production of extended-spectrum *β*-lactamases. *Clinical Infectious Diseases*.

[62] Lee SS, Chen YS, Tsai HC (2008). Predictors of septic metastatic infection and mortality among patients with *Klebsiella pneumoniae* liver abscess. *Clinical Infectious Diseases*.

[63] Cheng HP, Siu LK, Chang FY (2003). Extended-spectrum cephalosporin compared to cefazolin for treatment of *Klebsiella pneumoniae*-caused liver abscess. *Antimicrobial Agents and Chemotherapy*.

[64] Durand ML (2013). Endophthalmitis. *Clinical Microbiology and Infection*.

[65] Kashani AH, Eliott D (2013). The emergence of *Klebsiella pneumoniae* endogenous endophthalmitis in the USA basic and clinical advances. *Journal of Ophthalmic Inflammation and Infection*.

[66] Lee J, Oh CE, Choi EH, Lee HJ (2013). The impact of the increased use of piperacillin/tazobactam on the selection of antibiotic resistance among invasive *Escherichia coli* and *Klebsiella pneumoniae* isolates. *International Journal of Infectious Diseases*.

[67] Lee J, Pai H, Kim YK (2007). Control of extended-spectrum *β*-lactamase-producing *Escherichia coli* and *Klebsiella pneumoniae* in a children’s hospital by changing antimicrobial agent usage policy. *Journal of Antimicrobial Chemotherapy*.

[68] Tian LT, Yao K, Zhang XY (2012). Liver abscesses in adult patients with and without diabetes mellitus: an analysis of the clinical characteristics, features of the causative pathogens, outcomes and predictors of fatality: a report based on a large population, retrospective study in China. *Clinical Microbiology and Infection*.

[69] Ferraioli G, Garlaschelli A, Zanaboni D (2008). Percutaneous and surgical treatment of pyogenic liver abscesses: observation over a 21-year period in 148 patients. *Digestive and Liver Disease*.

[70] Porras-Ramirez G, Hernandez-Herrera MH, Porras-Hernandez JD (1995). Amebic hepatic abscess in children. *Journal of Pediatric Surgery*.

[71] Hope WW, Vrochides DV, Newcomb WL, Mayo-Smith WW, Iannitti DA (2008). Optimal treatment of hepatic abscess. *American Surgeon*.

[72] Onder A, Kapan M, Boyuk A (2011). Surgical management of pyogenic liver abscess. *European Review for Medical and Pharmacological Sciences*.

[73] Yeh FC, Yeh KM, Siu LK (2012). Increasing opsonizing and killing effect of serum from patients with recurrent K1 *Klebsiella pneumoniae* liver abscess. *Journal of Microbiology, Immunology and Infection*.

